# Bridging Hospital Resource Variability: Adapting the Escape Room to Integrate Procedure Teaching for Emergency Medicine Trainees in India

**DOI:** 10.21980/J8CK98

**Published:** 2024-10-31

**Authors:** Jodi DeJohn, Tania Ahluwalia, Manu Madhok, Shweta Gidwani, Katherine Douglass, Susan Owens

**Affiliations:** * University of Kentucky, Department of Emergency Medicine, Lexington, KY; ^ Children’s National Hospital, Department of Emergency Medicine, Washington, DC; † Children’s Minnesota, Department of Emergency Medicine, Minneapolis, MN; ** George Washington University, Department of Emergency Medicine, Washington, DC

## Abstract

**Audience:**

This is an in-person escape room and procedure simulation activity based on complications of human immunodeficiency virus (HIV) in India, which was created by using local HIV management guidelines. Emergency Medicine (EM) trainees of all post-graduate levels are the target audience. This may also be used by trainees in other specialties, such as infectious disease or internal medicine, who require an understanding of HIV and its complications. This escape room can be completed in teams of varying sizes and is designed to be adaptable to local resource availability.

**Background:**

Patients with HIV present to the Emergency Department (ED) for a variety of reasons such as initial viral syndrome, medication side effects, and opportunistic infections. While the widespread use of antiretroviral therapy (ART) has significantly increased the life expectancy of patients living with HIV and decreased the incidence of classical opportunistic infections, EM providers should still be vigilant and competent in diagnosing and managing these pathologies. This is particularly critical in India, where the prevalence of HIV was most recently estimated at 0.22% (2.2 million people older than 15 years) in 2020.^1^ This patient population, primarily infected through unprotected heterosexual contact, is at high risk for interruptions in ART and development of opportunistic infections for a variety of reasons including migration for work, low social status of women, and significant social stigma against HIV.^2^ Simulation is an educational opportunity to review these high-acuity low-occurrence presentations to prepare EM trainees for independent practice.

**Educational Objectives:**

By the end of the escape room, learners should be able to: 1) describe the mechanism of action of antiretroviral therapies available in India, 2) prescribe initial antiretroviral therapy to a patient presenting to the emergency department with a new diagnosis of HIV, 3) develop a differential diagnosis for a patient with HIV presenting to the ED with chest pain, 4) identify common dermatologic manifestations of opportunistic infections in patients with HIV, 5) identify computerized tomography scan and lumbar puncture features for central nervous system infections seen in patients with Acquired Immunodeficiency Syndrome (AIDS), 6) identify red flag features and appropriate workup for a patient with HIV presenting with a headache to the ED, 7) interpret images obtained during a Rapid Ultrasound for Shock and Hemorrhage (RUSH) exam, 8) identify cardiac tamponade and perform a pericardiocentesis, and 9) communicate and collaborate as a team to manage a complex, unstable patient with HIV in the ED.

**Educational Methods:**

We sought to create and implement an educational tool that could meet the complex education needs of EM trainees while being low cost, easily adapted to local resources, and engaging for trainees. Hospitals participating in the Masters in Emergency Medicine (MEM) program, a global partnership between the Ronald Reagan Institute for Emergency Medicine at the George Washington University and 18 hospitals in India, have resource variability for traditional simulation. The escape room created combines simulation, content review specific to the contextual practice of EM in India focused on HIV and its complications, and critical procedure teaching on pericardiocentesis. This innovation framework is based on Kolb’s experiential learning cycle and incorporates the gamification principles of a sense of autonomy, perception of competitiveness, and learner-relatedness.^3^–^4^ Escape rooms have been shown to engage learners, and low-fidelity procedure models could further maximize the experience for learners in resource variable settings.^5^ A pericardiocentesis model was adapted from Lord et al.’s low-fidelity model, ensuring it could be assembled with materials readily available in-country.^6^

**Research Methods:**

We adapted the escape room format to combine simulation, content review, and procedural training in a cost-effective, contextually relevant, and scalable way. The escape room was trialed using a case of chest pain and altered mental status caused by a pericardial effusion due to tuberculosis in a patient with HIV. Local practice patterns and guidelines were used to develop puzzles and clinical clues. A pericardiocentesis model was constructed using materials readily available in India. Pre- and post-surveys were developed to assess baseline trainee experience with escape rooms, self-reported knowledge of the differential diagnosis and management for altered mental status, and ways to incorporate escape room content into daily practice.

**Results:**

A total of 47 trainees participated; 41 of 47 participants completed both pre- and post-surveys (87% response rate). Participants represented all program trainee levels: 49% (n = 20) PGY-1, 27% (n = 11) PGY-2, and 24% (n = 10) PGY-3. Based on a score greater than seven on a 1–10 Likert scale, the escape room was rated as “highly effective” by 93.5% of respondents in reviewing medical knowledge. The trainees were allotted 60 minutes to escape the room; the median time for escape room completion was 57 minutes. The escape room and pericardiocentesis model cost under $100 USD, were repeated up to six times in one day, and could be recycled for future use.

**Discussion:**

Utilizing simulation in the escape room format that can be adaptable to variable resource settings is a valuable educational tool. The integrated escape room and procedure training proved to be an effective educational tool that was scalable and maintained efficacy across variable hospital resource levels. The next step includes adapting this format for other disease pathologies. This is a useful way to meet the education needs of MEM program trainees, regardless of hospital resource availability, that could be replicable in other EM training programs.

**Topics:**

HIV, AIDS, dermatologic manifestations of HIV, HIV medications, CNS complications of HIV, chest pain, headache, tuberculosis, RUSH exam, pericardiocentesis, escape room, simulation.

## USER GUIDE

[Table t1-jetem-9-4-sg24]

**Table t1-jetem-9-4-sg24:** 

List of Resources:	
Abstract	24
User Guide	27
Small Groups Learning Materials	31
Appendix A: Small Group Application Exercise with Answer Included with Each Puzzle/Clue	31
Appendix B: Brief Wrap Up	48

**Learner Audience:**

Interns, Junior Residents, Senior Residents, any healthcare provider who requires additional knowledge about HIV and its complications

**Time Required for Implementation:**

Preparation included 20 minutes to create the pericardiocentesis model using local materials, four hours to create and organize the escape room materials, one hour to develop a facilitator guide, and 15 minutes to set up the room. For the participants, the activity took a total of 85 minutes: five minutes for activity orientation, 60 minutes for the escape room, 10 minutes to debrief, and 10 minutes for pericardiocentesis practice.

**Recommended Number of Learners per Instructor**:

four to ten

**Topics:**

HIV, AIDS, dermatologic manifestations of HIV, HIV medications, CNS complications of HIV, chest pain, headache, tuberculosis, RUSH exam, pericardiocentesis, escape room, simulation.

**Objectives:**

By the end of this small group activity, learners will be able to:

1. Describe the mechanism of action of antiretroviral therapies available in India
2. Provide initial antiretroviral therapy to a patient presenting to the Emergency Department (ED) with a new diagnosis of HIV
3. Develop a differential diagnosis for a patient with HIV presenting to the ED with chest pain
4. Identify common dermatologic manifestations of opportunistic infections in patients with HIV
5. Identify computerized tomography (CT) scan and lumbar puncture (LP) features for central nervous system infections seen in patients with Acquired Immunodeficiency Syndrome (AIDS)
6. Identify red flag features and appropriate workup for a patient with HIV presenting with a headache to the ED
7. Interpret images obtained during a Rapid Ultrasound for Shock and Hemorrhage (RUSH) exam
8. Identify cardiac tamponade and perform a pericardiocentesis
9. Communicate and collaborate as a team to manage a complex, unstable patient with HIV in the ED

### Linked objectives and methods

The practice of Emergency Medicine (EM) requires synthesis of the practitioner’s knowledge, procedural skill, decision-making capacity, and mastery of interpersonal dynamics to lead an effective team; these complex learning needs are addressed well with simulation.^7^ Escape rooms combine multiple puzzles for collaboration among team members to complete. They have been used in medical education as an active form of learning to incorporate decision-making and teamwork and have been successfully piloted among EM trainees.^8^ The escape room format in the US has been used for EM skills such as research, patient safety, trauma procedures, and ultrasound.^9^–^12^ Escape rooms have been shown to engage learners, and low-fidelity procedure models could further maximize the experience for learners in resource variable settings.^5^

This project occurred during the infectious disease module of the three-year EM curriculum. A clinical scenario was developed as the framework for the escape room relevant to EM in India: a patient with HIV presenting with chest pain and altered mental status (AMS) caused by pericardial effusion due to tuberculosis. Within this clinical scenario, escape room educators identified learning objectives based on the core knowledge needed to manage the case. These objectives are linked to the case’s progression using various puzzles placed throughout the room. Indian national guidelines for treating HIV and opportunistic infections made the case contextually relevant.^1^ The escape room was designed to readily adapt to site resources, including simulation mannequin fidelity and physical space.

A pericardiocentesis model was adapted from Lord et al.’s low-fidelity model, ensuring it could be assembled with materials readily available in-country.^6^ The adapted low-fidelity pericardiocentesis model was easily reproduced from supplies readily available in India using balloons, food coloring, masking tape, a large rectangular basin, waterproof transparent dressings, poster board, binder clips, and a yoga mat. Pre- and post-surveys were developed to assess baseline trainee experience with escape rooms, self-reported knowledge of the differential diagnosis and management for altered mental status, and ways to incorporate escape room content into daily practice.

As the team enters the room, there is introductory case content on the simulation mannequin stating that the patient presented with altered mental status and chest pain with associated hypotension and hypoxia. Two puzzle sequences will ultimately provide the team with enough information to escape the room.

#### Educational Objective (EO) 1

Describe the mechanism of action of antiretroviral therapies available in India. This starts the first puzzle sequence. Using a deck of cards, the team must sort medications used to treat HIV by drug class (reverse transcriptase inhibitor, protease inhibitor, integrase inhibitor, and CCR5 Entry Inhibitors). Each drug class is assigned a number, and all drugs in the same class are on the same suit of playing cards (heart, diamond, spade, club). A key card has the card suits displayed in the order of the four-digit combination needed to open a four-digit lock that contains three small colored keys and the puzzle for EO 2.

#### EO 2

Prescribe initiation antiretroviral therapy to a patient presenting to ED with new diagnosis of HIV. This is a multiple-choice question asking about initial antiviral therapy for a patient who has tested positive for HIV in the Emergency room. The answers are in colored font that corresponds to three colored keys in the room. Selecting the correct response/key will open another lock containing the next puzzle for EO 3. The other two keys will open locks that do not contain puzzles.

#### EO 3

Develop a differential diagnosis for a patient with HIV presenting to the ED with chest pain. This is a crossword puzzle with clues and a word list. The team must complete the crossword correctly to access the key. The key opens a lock containing a blank card with invisible ink that reads, “Perform a pericardiocentesis to save your patient and escape the room.”

#### EO 4

Identify common dermatologic manifestations of opportunistic infections in patients with HIV. This starts the second puzzle sequence. Taped to various surfaces around the room are pictures of dermatologic manifestations of HIV and opportunistic infections glued to colored paper and their diagnoses.

A “key” containing three colored boxes was taped to the wall in the order of the code needed to open the next lock. Each diagnosis was associated with a number. The team must match up the image with diagnosis to reveal the code for a 3-number rotary combination lock. Opening the rotary lock will reveal the puzzle for EO 5.

#### EO 5

Identify CT scan and LP features seen in central nervous system infections in patients with AIDS. This is a matching activity in which the team must match the diagnosis with its associated CT head and LP results. Each diagnosis is associated with a letter. The diagnoses must be in the correct order to reveal the 5-letter code for the alphabetical lock. This puzzle activity also contains relevant clinical information to the simulation case: a chest x-ray showing miliary tuberculosis and a venous blood gas indicating respiratory acidosis. Opening the alphabetical lock will reveal the puzzle for EO 6.

#### EO 6

Identify red flag features and appropriate workup for a patient with HIV presenting with headache to the ED. This is a word search puzzle with seven terms relating to red-flag features of headaches in patients with HIV. Completing the word search will provide access to an orange key that opens an orange lock containing an invisible ink light.

#### EO7

Interpret images obtained during a RUSH exam. During the puzzle for EO6, teams will also be instructed to conduct an ultrasound on the hypotensive patient. Teams should request (or perform, based on resource availability and mannequin fidelity) a RUSH exam. The team interprets the images revealing a large pericardial effusion.

#### EO8

Identify pericardial tamponade and perform a pericardiocentesis. After completing both puzzle sequences, the teams will discover that the special pen light reveals final instructions to escape the room. Upon escaping the room, teams will then review the steps and practice a pericardiocentesis on a low-fidelity model.

### Recommended pre-reading for facilitator

The facilitator guide details how the case and puzzles should progress. Background reading and review can be done with the following resources:

1. Marco CA, Balhara KS, Rothman RE. Human immunodeficiency virus infection. In: Tintinalli JE, Ma O, Yealy DM, et al, eds. *Tintinalli’s Emergency Medicine: A Comprehensive Study Guide, 9e*. McGraw Hill; 2020. Accessed December 15, 2023. At: https://accessemergencymedicine.mhmedical.com/content.aspx?bookid=2353&sectionid=220292558
2. National AIDS Control Organization. National guidelines for HIV care and treatment, 2021. Ministry of Health and Family Welfare, Government of India. (accessed on July 18 2022 at: https://naco.gov.in/sites/default/files/National_Guidelines_for_HIV_Care_and_Treatment_2021.pdf)
3. Evaluation office, UNAIDS. Evaluation of UNAIDS joint Programme Country Envelopes: 2018 – 2022, case study: India. UNAIDS. (accessed 8 December 2023 at: https://www.unaids.org/sites/default/files/media/documents/evaluation-country-envelopes-2018-2022-case-study-india_en.pdf
  It would be appropriate to substitute country-specific HIV guidelines (resource 2 and 3) based on geographic context.

### Learner Responsible Content (LRC)

1. Marco CA, Balhara KS, Rothman RE. Human immunodeficiency virus infection. In: Tintinalli JE, Ma O, Yealy DM, et al, eds. *Tintinalli’s Emergency Medicine: A Comprehensive Study Guide, 9e*. McGraw Hill; 2020. Accessed December 15, 2023. At: https://accessemergencymedicine.mhmedical.com/content.aspx?bookid=2353&sectionid=220292558
2. National AIDS Control Organization. National guidelines for HIV care and treatment, 2021. Ministry of Health and Family Welfare, Government of India. (accessed on July 18 2022 at: https://naco.gov.in/sites/default/files/National_Guidelines_for_HIV_Care_and_Treatment_2021.pdf)
  1. This reference can be adapted to geographic context.

### Small group application exercise (sGAE)

Attached as Appendix A

### Results and tips for successful implementation

**Figure 1:**
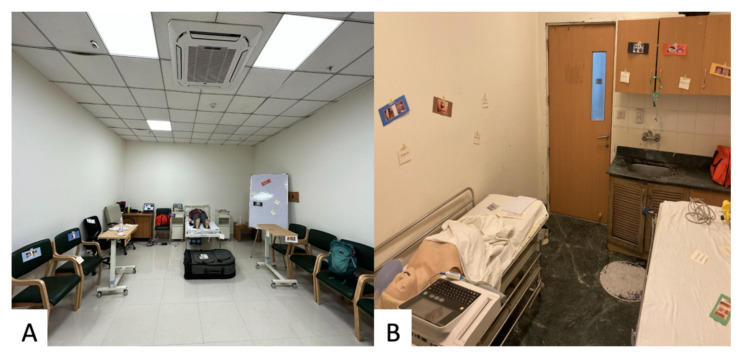
Escape Room Set-Up in Delhi (A) and Dehradun (B)

A total of 47 trainees participated from Delhi (n=32), Dehradun (n=14), and Kolkata (n=4) involving residents from 6 hospitals. Participants represented all program trainee levels: 49% (n = 20) PGY-1, 27% (n = 11) PGY-2, and 24% (n = 10) PGY-3. Trainees were grouped into teams of 4–12; the number of participants varied based on site, though ideally a maximum of ten participants should be in each small group to ensure participation. Trainees from four hospital sites in Delhi participated in the escape room at a simulation training center with a high-fidelity mannequin. Two additional sites participated in the escape room held in an ED patient room and a classroom with a half-torso mannequin, congruent with how trainees routinely perform simulations at their sites. A pre-briefing included an explanation of the 60-minute time limit, the general puzzle format, and the availability of clinical and logistical clues if needed. Trainees completed a pre-survey regarding their experience with escape rooms and HIV management. Regardless of location, a mannequin was placed in the center of a closed room with a folder containing basic patient information and surrounded by multiple puzzles (see Figure 1). Decompensating vitals were delivered using the simulation mannequin technology or an iPad throughout the case depending on the site’s resources.

The simulation case ran continuously as the small group collaborated to solve puzzles based on learning objectives supplemented with evolving case content. Ultimately, the patient decompensates with the final puzzle solution, leading the learner to interpret a rapid ultrasound for shock and hypotension (RUSH) exam. The RUSH exam reveals pericardial tamponade, and the team must state the emergent intervention, a pericardiocentesis, to escape the room.

After the activity, a debrief was conducted using the promoting excellence and reflective learning in simulation (PEARLS) framework.^13^ During the debriefing, the objectives and solutions of each puzzle were discussed; afterward, trainees were invited to practice pericardiocentesis using the low-fidelity model.

Trainees were asked to complete a post-survey about their experience. Of the 47 participants, 41 participants completed both the pre- and post-surveys (87% response rate). Based on a score greater than seven on a 1–10 Likert scale, 93.5% of respondents rated the escape room as “highly effective” in reviewing medical knowledge When evaluated by trainee level, average Likert score values regarding effectiveness of the escape room as a method for content review were 9.42 for PGY-1, 9.13 for PGY-2, and 9.92 for PGY-3. Free-text survey responses revealed excitement for the combination format, an appreciation for teamwork practice, and the realism added by time pressure. The median time to “escape” was 57 minutes, comparable to the typical length of a visiting faculty didactic session. Suggestions for improvement included a more detailed debrief and standardized use of hints.

This innovation is low-cost with materials for the escape room, primarily the locks and printed puzzles and the pericardiocentesis model, costing less than $100 USD. This escape room successfully demonstrated the adaptability of this innovation across sites with various simulation capabilities, from interactive mannequins to silicone torsos, and variability in space, from dedicated classrooms to empty patient rooms in the ED. None of the sites had commercial pericardiocentesis procedure models, and the participants were eager to practice a procedure previously only read about. The adapted low-fidelity pericardiocentesis model was easily reproduced from supplies readily available in India using balloons, food coloring, masking tape, a large rectangular basin, waterproof transparent dressings, poster board, binder clips, and a yoga mat. This escape room could be further adapted to different geographical contexts with simple changes to puzzle content, such as skin tones on the dermatologic manifestations, and the use of local HIV guidelines for treatment initiation.

This combined format, including simulation, an escape room, and a procedural skill station, is an efficient use of time that benefits from the experiential learning theory and maintains learners’ self-reported efficacy across different hospital resource levels. Feedback was overwhelmingly positive, showing the residents enjoyed this format for content review and procedural teaching. This project satisfies level one of the Kirkpatrick model; future work will be done to evaluate the ongoing impact of this escape room activity.

### Associated Content

Appendix A: Facilitator Guide

Appendix B: Brief Wrap Up

## Figures and Tables

**Figure f4-jetem-9-4-sg24:**
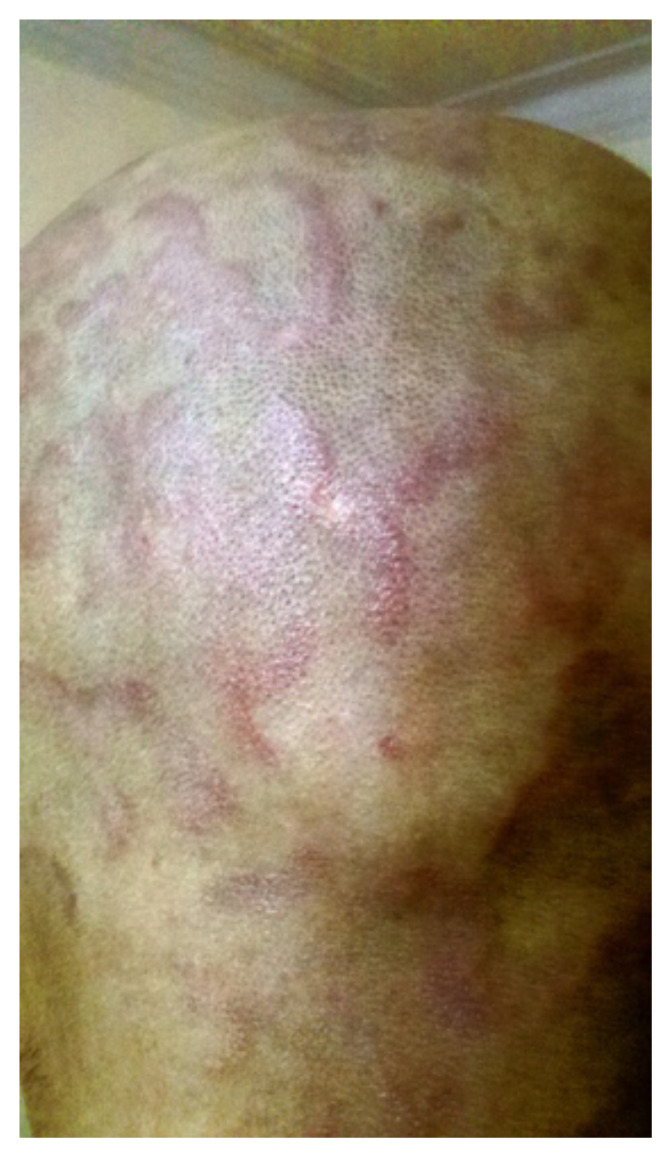
Seborrhoeic dermatitis. In: File:Seborrhoeic Dermatitis on the Scalp.jpg [web]. https://commons.wikimedia.org/wiki/File:Seborrhoeic_Dermatitis_on_the_Scalp.jpg. 2024. CC BY-SA 3.0.

**Figure f5-jetem-9-4-sg24:**
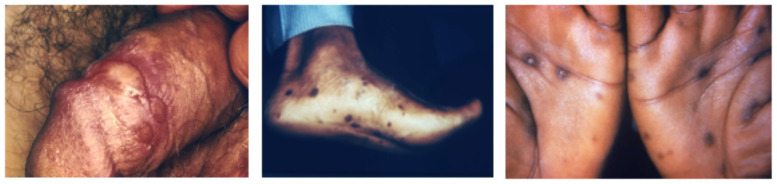
Fiumara N. Syphilis. 1976. CDC Public Health Image Library. https://phil.cdc.gov/Details.aspx?pid=17855. Public domain. Lindsley L. Syphilis. 1972. CDC Public Health Image Library. https://phil.cdc.gov/Details.aspx?pid=3971. Public domain. Syphilis. 1971. CDC Public Health Image Library. https://phil.cdc.gov/Details.aspx?pid=17043. Public domain.

**Figure f6-jetem-9-4-sg24:**
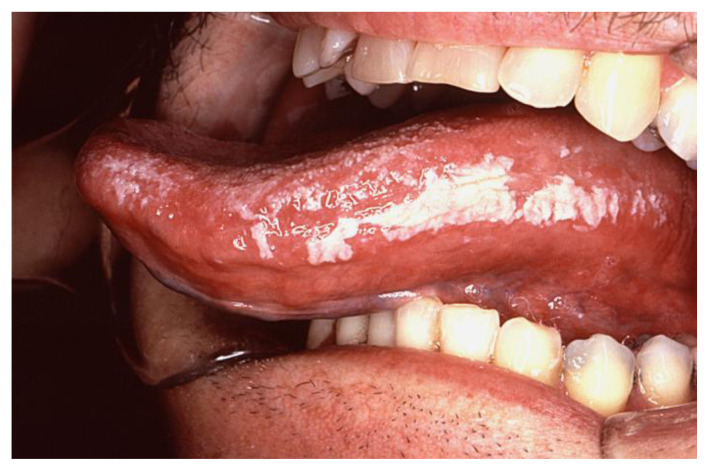
Silverman S. Oral Hairy Leukoplakia.1987. CDC Public Health Image Library. https://phil.cdc.gov/Details.aspx?pid=6060. Public Domain.

**Figure f7-jetem-9-4-sg24:**
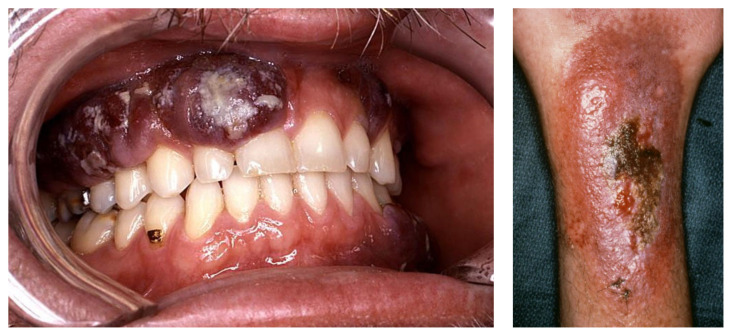
Silverman S. Kaposi’s Sarcoma. 1999. CDC Public Health Image Library. https://phil.cdc.gov/Details.aspx?pid=6058. Public domain. Kraus S. Kaposi’s Sarcoma. 1981. CDC Public Health Image Library. https://phil.cdc.gov/Details.aspx?pid=5514. Public domain.

**Figure f8-jetem-9-4-sg24:**
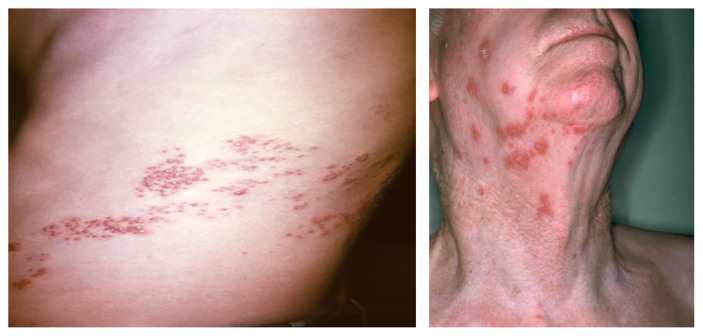
Hermann, K. Herpes Zoster. 1966. CDC Public Health Image Library. https://phil.cdc.gov/Details.aspx?pid=21506. Public domain. National Institute of Allergy and Infectious Diseases. Herpes Zoster. 2012. CDC Public Health Image Library. https://phil.cdc.gov/Details.aspx?pid=18254. Public Domain.

**Figure f9-jetem-9-4-sg24:**
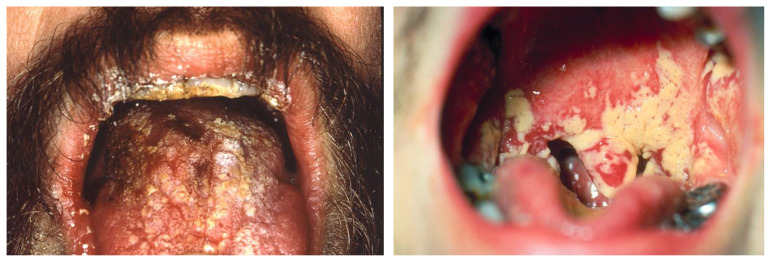
Molinari J, Silverman S. Oral Candidiasis. 1987. CDC Public Health Image Library. https://phil.cdc.gov/Details.aspx?pid=6067. Public domain. Silverman S. Pseudomembranous Candidiasis. 1987. CDC Public Health Image Library. https://phil.cdc.gov/Details.aspx?pid=6066. Public domain.

**Figure f10-jetem-9-4-sg24:**
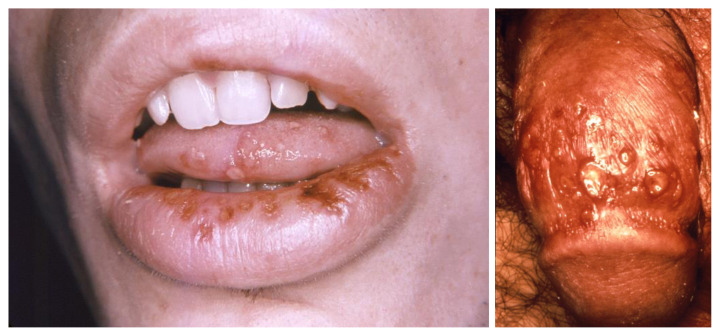
Sumpter R. Oral Herpes. 1967. CDC Public Health Image Library. https://phil.cdc.gov/Details.aspx?pid=12616. Public domain. Flumara N, Hart G. Genital Herpes. 1976. CDC Public Health Image Library. https://phil.cdc.gov/Details.aspx?pid=6471. Public domain.

**Figure f11-jetem-9-4-sg24:**
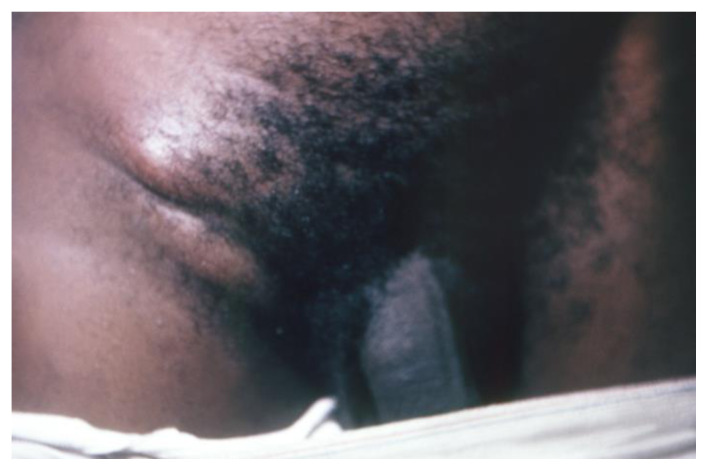
Woodall R. Lymphogranuloma Venereum. 1969. CDC Public Health Image Library. https://phil.cdc.gov/Details.aspx?pid=18038. Public domain.

**Figure f12-jetem-9-4-sg24:**
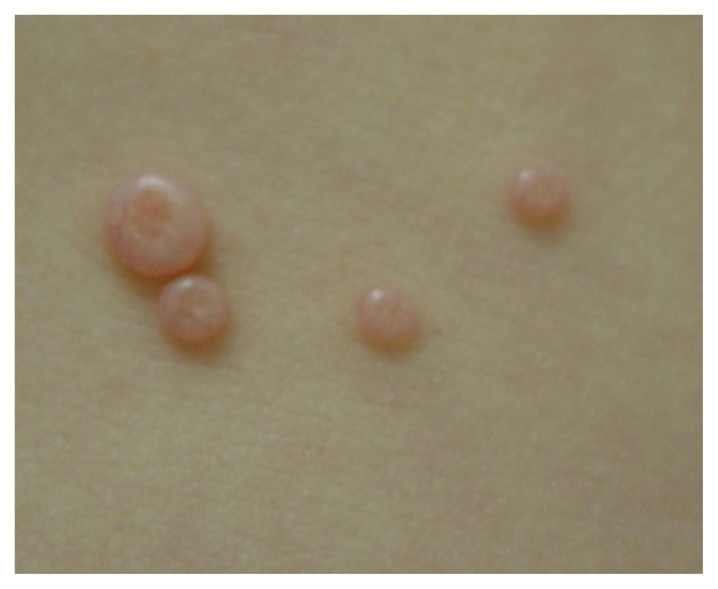
Bray W. Molluscum Contagiosum. 2011. https://commons.wikimedia.org/wiki/File:Molluscum_bumps.jpg. Public Domain.

**Figure f13-jetem-9-4-sg24:**
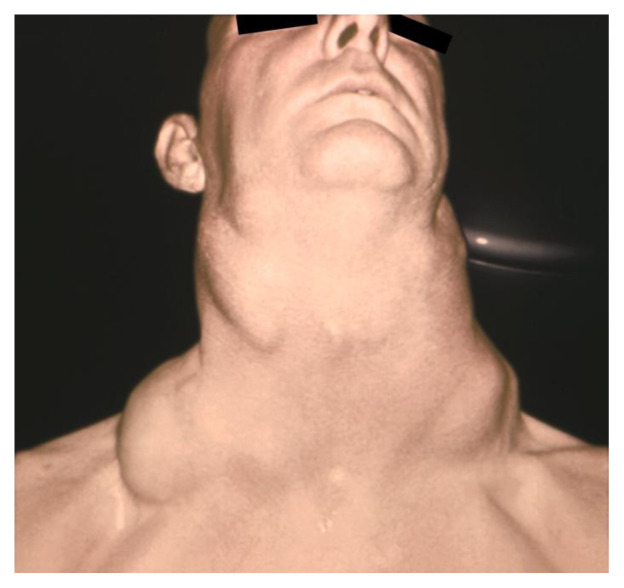
Sumpter R. Lymphoma. 1967. CDC Public Health Image Library. https://phil.cdc.gov/Details.aspx?pid=12630. Public domain.

**Figure f17-jetem-9-4-sg24:**
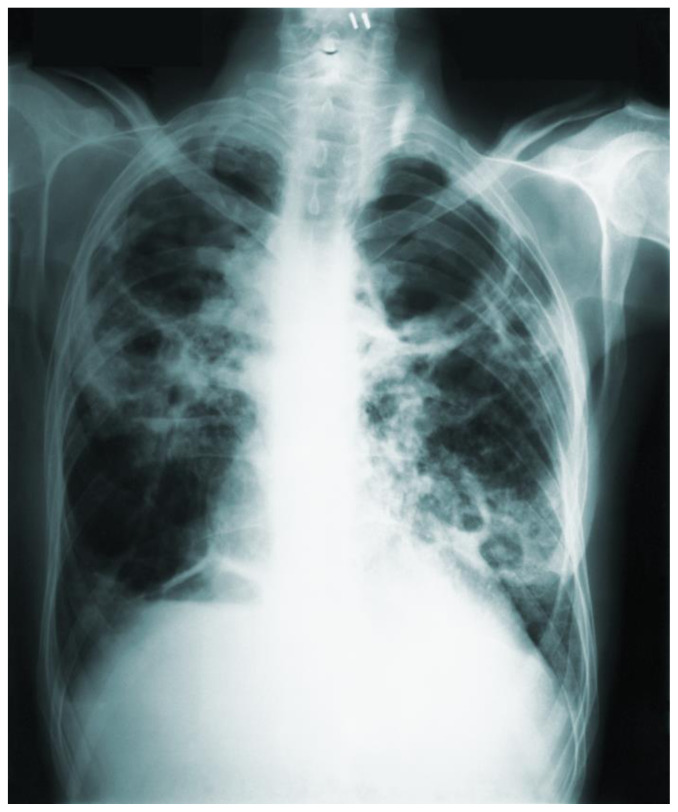
Larkin R. Tuberculosis. 1966. CDC Public Health Image Library. https://phil.cdc.gov/Details.aspx?pid=20118. Public domain.

## SMALL GROUPS LEARNING MATERIALS

### Appendix A Small Group Application Exercise (sGAE) with Answers Included with Each Puzzle/Clue

**Stations:**

**Patient Narrative (laying on mannequin):**

A 30 yo man presents via EMS with altered mental status and chest pain. Patient is cachectic, GCS (Glasgow Coma Score) 12, BP: 90/60, SpO2 90% on room air, HR 112, RR 22. Solve the puzzles in the room to learn more information about your patient and discover his diagnosis in order to escape the breakout room.

**Clue 1a:** Playing Cards

*Learning Objective:* Describe mechanism of action of antiretroviral therapies available in India (EO1).

*Game Objective:* group the playing cards by drug class

*Answer:* 2-7-8-5 (for digit lock)[Table t2-jetem-9-4-sg24]

**Table t2-jetem-9-4-sg24:**

Reverse Transcriptase Inhibitors: hearts (2)
Zidovudine
Lamivudine
Abacavir
Nevirapine
Efavirenz
Tenofovir
Protease Inhibitors: clubs (7)
Ritonavir
Atazanavir
Lopinavir
Darunavir
Integrase Inhibitors: spades (8)
Dolutegravir
Raltegravir
CCR5 Entry Inhibitors: diamonds (5)
Maraviroc

**Clue 1b:** Dermatologic Manifestations of HIV

*Learning Objective:* Identify common dermatologic manifestations of opportunistic infections in patients with HIV (EO 4).

*Game Objective:* match the disease photo with the disease name

*Answer:* 18-08-30 (three digit rotary lock)[Fig f3-jetem-9-4-sg24]

Seborrheic dermatitis-brown-32[Fig f4-jetem-9-4-sg24]

Syphilis-green-08[Fig f5-jetem-9-4-sg24]

Oral hairy leukoplakia-purple-10[Fig f6-jetem-9-4-sg24]

Kaposi sarcoma-light blue-22[Fig f7-jetem-9-4-sg24]

Shingles-white-05[Fig f8-jetem-9-4-sg24]

Candidiasis-orange-16[Fig f9-jetem-9-4-sg24]

HSV-red-30[Fig f10-jetem-9-4-sg24]

Lymphogranuloma Venereum -dark blue-18[Fig f11-jetem-9-4-sg24]

Musculosum-black-28[Fig f12-jetem-9-4-sg24]

Non-Hodgkin’s Lymphoma -yellow-03[Fig f13-jetem-9-4-sg24]

**Clue 2a:** Multiple Choice Question

*Learning Objective:* Provide initiation antiretroviral therapy to a patient presenting to ED with new diagnosis of HIV (EO 2).

*Game Objective:* choose the correct color to open the correct lock

*Answer:* pink

Distractors: yellow and blue locks that open containers without clues

Question:

A 36 yo M presents to the ED with two weeks of malaise, fever, chills, and decreased oral intake. Further history reveals patient has history of IV drug use and chronic kidney disease (CKD). You screen patient for HIV and Hep C and patient’s antibody/antigen HIV screening is positive for HIV. After further workup, what initial ART would be an appropriate regimen for this patient?

**Clue 2b:** Central Nervous System (CNS) Manifestations of Opportunistic Infections

*Learning Objective:* Identify computerized tomography (CT) scan and lumbar puncture (LP) features in central nervous system disease in AIDS patients.

*Game Objective:* match the disease name with CT and LP findings

*Answer:* DPEOY[Table t3-jetem-9-4-sg24]

**Table t3-jetem-9-4-sg24:**

CNS Disease	CT Head findings	LP
D: Toxoplasmosis	Ring-enhancing lesions	Lymphocytic
P: Primary CNS lymphoma	Homogeneous enhancing mass	Lymphocytic
E: Progressive Multifocal Leukoencephalopathy	Non-enhancing white matter lesions	JC virus by PCR Quantitative
O: Cryptococcal meningitis	hydrocephalus/normal	Elevated opening pressure, cerebral spinal fluid (CSF) CrAg + (cryptococcal antigen)
Y: Tuberculosis meningitis	Normal	Elevated opening pressure

*Clinical information included in box along with puzzle:* A chest xray showing miliary tuberculosis and venous blood gas (VBG) showing hypercapnic and hypoxic respiratory failure

VBG:

pH: 7.25

PO2: 35

PCO2: 65

HCO3: 20

Glucose: 80[Fig f17-jetem-9-4-sg24]

**Clue 3a:** Crossword Puzzle

*Learning Objective:* Develop a differential diagnosis for a patient with HIV presenting to the ED with chest pain.

*Game Objective:* complete crossword to access red key

- Red key contains envelope with “blank paper” that reads, “Perform a pericardiocentesis to save your patient and escape the room,” only visible with ink light

**Crossword Puzzle Key:**

**Across:**

5. Prothrombotic state increases risk – **pulmonary embolism**
6. Caused directly by HIV and nutritional deficiencies, decreases lifespan by 75% - **dilated cardiomyopathy**
7. Associated with IV drug use, embolization of disease - **endocarditis**
8. Gradual, cough, fever, night sweats, weight loss, and lymphadenopathy; CXR with alveolar and miliary opacities at low CD4 counts versus cavitary lesions at higher CD4 counts - **tuberculosis**
9. CD4 < 200, elevated LDH, bilateral granular opacities on CXR, gradual onset, nonproductive cough – **PCP pneumonia**

**Down:**

1. Most common form of cardiac involvement, etiologies include KS, mycobacteria, CMV, bacteria, lymphoma, can be asymptomatic or deadly – **pericardial effusion**
2. Most often caused by candida, associated with odynophagia and dysphagia - **esophagitis**
3. Most common etiologies include Streptococcus pneumonia and Haemophilus Flu, cough with purulent sputum – **bacterial pneumonia**
4. Chronic inflammation and immune activation accelerate progression of cholesterol plaque erosion and rupture – **coronary artery disease**

**What is your differential for Chest pain in HIV + patient?** [Fig f18-jetem-9-4-sg24] [Table t4-jetem-9-4-sg24]

**Table t4-jetem-9-4-sg24:**

Across:	Down:
5. Prothrombotic state increases risk 6. Caused directly by HIV and nutritional deficiencies, decreases lifespan by 75% 7. Associated with IV drug use, embolization of disease 8. Gradual, cough, fever, night sweats, weight loss, and lymphadenopathy; CXR with alveolar and miliary opacities at low CD4 counts versus cavitary lesions at higher CD4 counts 9. CD4 < 200, elevated LDH, bilateral granular opacities on CXR, gradual onset, nonproductive cough	Most common form of cardiac involvement, etiologies include KS, mycobacteria, CMV, bacteria, lymphoma, can be asymptomatic or deadly Most often caused by candida, associated with odynophagia and dysphagia Most common etiologies include S. pneumonia and H. Flu, cough with purulent sputum Chronic inflammation and immune activation accelerate progression of cholesterol plaque erosion and rupture

**Word Bank:**

Viral pneumonia

Esophagitis

Myocardial infarction

Aortic dissection

Pleural effusion

Restrictive cardiomyopathy

Dilated cardiomyopathy

Heart failure

Sepsis

Restrictive lung disease

Bacterial pneumonia

Pericardial effusion

Pericardial tamponade

Pulmonary hypertension

Pulmonary embolism

Pneumocystis (PCP) pneumonia

Endocarditis

Coronary artery disease

Hypertension

Gastroesophageal Reflux

Aortic aneurysm

Tuberculosis

**Clue 3b:** Word Search

*Learning Objective:* Identify red flag features and appropriate workup for HIV patient presenting with headaches to the ED.

*Game Objective:* complete word search to access orange key

Word Search Puzzle Key:[Fig f19-jetem-9-4-sg24]

Find the alarm symptoms for an HIV + patient presenting with a headache and the diagnostic studies that should be considered for a patient presenting with these symptoms to access orange key.[Fig f20-jetem-9-4-sg24]

Clinical content included in box with puzzle: card instructing learner to perform a RUSH exam on your undifferentiated hypotensive patient:

- views displayed on iPad as the team places probe in correct position
- views: “HIMAP”: subxiphoid/cardiac with tamponade, plethoric inferior vena cava (IVC), Morrison’s pouch - normal, aorta - normal, pulmonary - normal lung sliding without B lines

Orange key: contains an invisible ink light to be able to read the “blank paper.”

#### RUSH Exam Video Clips

Avila, J. The RUSH Exam. Core Ultrasound. April 27 2021. Accessed Jan 1 2023. At: https://coreultrasound.com/rush-exam/

##### Cardiac View

Clip Bank (Cardiac): Tamponade – Subxiphoid. Core Ultrasound. March 4 2019. Accessed January 3 2023. At: https://coreultrasound.com/cb_cardiac/

##### Inferior Vena Cava (IVC)

Inferior Vena Cava Ultrasound: Plethoric IVC Clip. WikiEM. Aug 30 2020. Accessed Jan 3 2023. At: https://wikem.org/wiki/IVC_ultrasound

##### Morrison’s Pouch

Clip Bank (FAST Exam): RUQ-Normal. Core Ultrasound. March 24 2019. Accessed Jan 3 2023. At: https://coreultrasound.com/cb_fast/

##### Aorta

Clip Bank (Aorta): Normal, transverse. Core Ultrasound. Jan 16 2019. Accessed Jan 3 2023. At: https://coreultrasound.com/aorta_cb/

##### Pulmonary

Clip Bank (Lungs): Lung Sliding. Core Ultrasound. March 24 2019. Accessed Jan 3 2023. At: https://coreultrasound.com/lungs_cb/

### Appendix B Brief Wrap Up

#### HIV in the Emergency Department

##### State of HIV in India

National AIDS Control Organization & ICMR-National Institute of Medical Statistics (2022). India HIV Estimates 2021: Fact Sheet. New Delhi: NACO, Ministry of Health and Family Welfare, Government of India.

**should be adapted to national guidelines based on geographic context[Fig f21-jetem-9-4-sg24]

##### An Overview on HIV

Long, B. emDOCs Podcast – Episode 46: HIV. emDocs.com. Feb1, 2022. Accessed Jan 2023. Includes notes and brief podcast at: http://www.emdocs.net/emdocs-podcast-episode-46-hiv/[Fig f22-jetem-9-4-sg24]

##### Tuberculosis and HIV: WHO Guidelines

World Health Organization. Tuberculosis and HIV. Who.int. Accessed Jan 2023. At: https://www.who.int/teams/global-hiv-hepatitis-and-stisprogrammes/hiv/treatment/tuberculosis-hiv[Fig f23-jetem-9-4-sg24]

##### Pericardiocentesis: Overview, Procedural Review, and Expert Commentary

Feiger, D. Ibiebele, A. Pericardiocentesis. NUEM Blog. Feb 8, 2021. Accessed Jan 2023. At: http://www.nuemblog.com/blog/pericardiocentesis[Fig f24-jetem-9-4-sg24]
